# Pneumatosis cystoides intestinalis revealed after a hand-to-hand aggression: A case report

**DOI:** 10.1016/j.ijscr.2019.07.071

**Published:** 2019-08-01

**Authors:** A. Belkhir, M. Jrad, A. Sebei, M. Soudani, A. Haddad, S. Boukriba, W. Frikha, H. Mizouni

**Affiliations:** a Department of Radiology La Rabta University Hospital, Tunis, Tunisia; b Department of Surgery La Rabta University Hospital, Tunis, Tunisia

**Keywords:** Pneumatosis cystoides intestinalis, Pneumoperitoneum, Case report, CT imaging, Therapy

## Abstract

**Introduction:**

Pneumatosis cystoides intestinalis (PCI) is a condition defined by the presence of multiple gas-filled cysts within the intestinal wall. We demonstrated a case of PCI presenting as pneumoperitoneum following a hand-to-hand aggression. Consent was obtained from the patient for publication of this paper.

**Presentation of the case:**

This article describes a case of an 28 year-old man with medical history of gastroduodenal ulcer admitted in the emergency room with an acute abdominal pain secondary to a hand-to-hand aggression. Computed tomography (CT-scan) revealed signs of PCI, the presence of pneumoperitoneum and a small amount of fluid in the Douglas pouch. The patient underwent an urgent laparotomy in front of a high suspicion of a bowel perforation. Per operative findings revealed multiple small cysts of the terminal ileum and there were no bowel perforation.

**Discussion:**

Pneumatosis cystoides intestinalis may be related to a wide spectrum of gastrointestinal conditions. The diagnosis of PCI can be established by endoscopic ultrasound or CT-scan imaging. Management of PCI is conditioned by the clinical and radiological presentation which is essentially related to the primary cause. Conservative approach is allowed in a stable patient with no signs of complications. In the presence of predictive factors of pathologic PCI, namely transmural ischemia and bowel perforation, surgical operation is required.

**Conclusion:**

The management of PCI may be challenging particularly in the presence of pneumoperitoneum. Complications must be excluded before considering a conservative therapy. Therefore, PCI should be interpreted with relevance to the entire clinical context.

## Highlights

• Pneumatosis cystoides intestinalis should be interpreted with relevance to the entire clinical context.
• Positive diagnosis can be established by computed tomography imaging.
• Management of PCI is conditioned by the clinical and radiological presentation which is essentially related to the primary cause.
• Surgical procedure is required when suspecting transmural ischemia or bowel perforation.

## 1 Introduction

Pneumatosis cystoides intestinalis (PCI) is not a disease but a condition characterized by the presence of multiple gas-filled cysts within the submucosa or subserosa layer of the intestinal wall. Its clinical significance may vary from ischemic origin to a benign finding. Since the broad indications of computed tomography (CT-scan), the incidence of pneumatosis intestinalis is becoming more common than previously reported. At the present time, there are no consensus to guide surgical intervention.

Here, we present a case of a PCI revealed by pneumoperitoneum following a hand-to-hand aggression, in a 28 year-old man. Consent was obtained from the patient in question for publication of this case report and accompanying images. A copy of the written consent is available upon request. Our work has been reported in ligne with SCARE criteria [1].

## 2 Presentation of the case

**Fig. 1:**
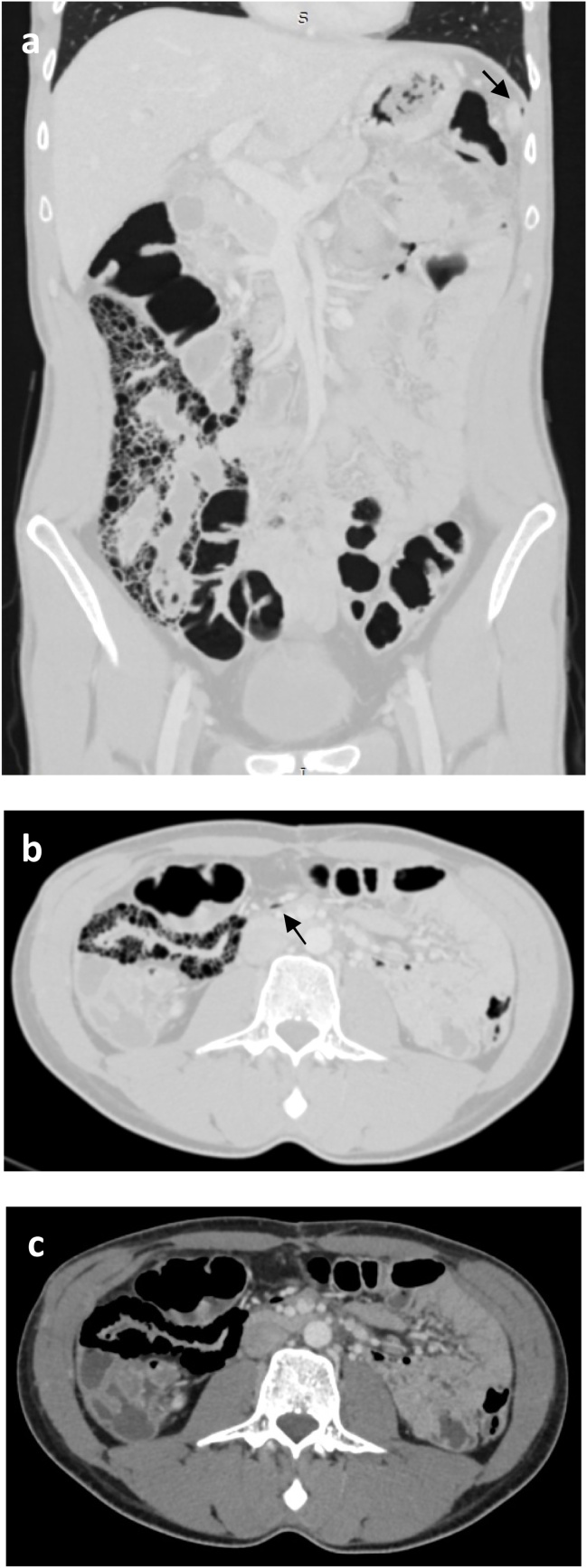
Portal enhanced CT-scan of the abdomen and pelvis: Coronal and axial views in lung window setting (a, b) and axial view in soft tissus window setting (c) showing gas in multiple small cysts within the wall of the terminal ileum. Presence of pneumoperitoneum under the left hemi diaphragm and within the mesenter (arrowheads).

A 28 year-old man with medical history of gastroduodenal ulcer admitted to the emergency room with an acute abdominal pain secondary to a hand-to-hand aggression. Vital signs were normal. Physical examination showed a diffuse abdominal tenderness with normal bowel sounds. No abdominal distension or contracture were noticed. The patient was apyretic. Laboratory investigations were normal. Initially, the patient was explored by abdominal sonography which revealed a small amount of anechoic fluid in the Douglas pouch. However, the exploration of the solid organs was no conclusive because of the interposition of a gas screen. Thus, an abdominopelvic CT-scan was performed revealing multiple small gas-filled cysts within the wall of the terminal ileum, the presence of pneumoperitoneum and a small amount of hypodense fluid in the Douglas pouch (Fig. 1). No other abnormalities were seen.

**Fig. 2:**
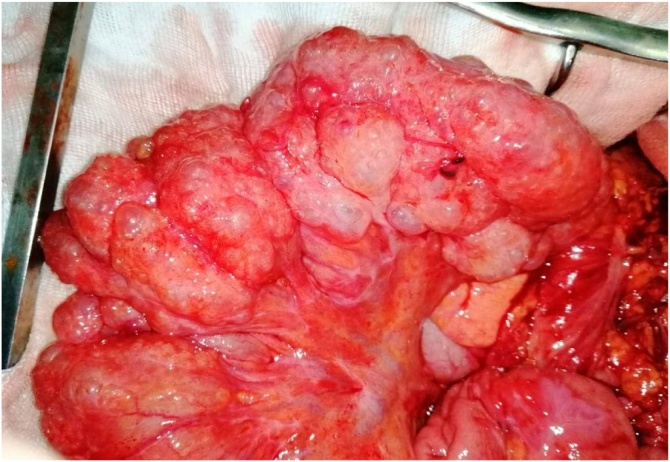
Picture of the operative field demonstrating pneumatosis cystoides intestinalis within the terminal ileum.

The patient underwent an urgent laparotomy, in front of a high suspicion of a bowel perforation. Per operative findings revealed multiple small cysts of the terminal ileum and there were no bowel perforation (Fig. 2). The postoperative period was uneventful.

## 3 Discussion

Pneumatosis cystoides intestinalis tends to occur more frequently in the colon followed by the small intestine. Combined involvement was noticed in few cases [2]. Location confined to small intestine was correlated with the presence of an underlying bowel disease (such as bowel perforation, bowel infarct, enteritis, bowel obstruction) (3). In the present case, the terminal ileum was affected. The rest of the bowel tract was normal.

Although the pathophysiology is incompletely understood, two main theories exist [3,4]. The mechanical theory suggests that intestinal mucosa injury allows normal gas to dissect into the bowel wall, especially under increased intraluminal pressure. The bacterial theory postulates that gas-forming bacilli enter the submucosa through mucosal breachs or increased mucosal permeability and subsequently form gas within the bowel wall [4].

PCI may be related to a wide spectrum of gastrointestinal conditions including peptic ulcer, inflammatory bowel disease, intestinal obstruction, and intestinal necrosis, as well as, endoscopic procedures and post abdominal surgery. Connective tissue anomalies and pulmonary disease were seen too [2]. It may be also associated with chemotherapy and steroid therapy [3].

Most PCI are asymptomatic and incidentally detected on CT or during surgery. Symptoms vary depending on the underlying cause and may consist of abdominal pain, diarrhea, distention, nausea and vomiting, bloody stool, mucous stool or constipation [2].

CT-scan is a sensitive method for detecting PCI. The use of a lung window setting is the key of the diagnosis. Gas cysts in the bowel wall may not be apparent at the soft tissue window setting. Furthermore, CT-scan allows the detection of combined pathological conditions participating in the decision-making process [3,5,6]. Lee et al demonstrated that mesenteric stranding, bowel wall thickening and ascites were CT-scan features of clinically worrisome PCI heralding underlying bowel disease. Pneumoperitoneum was found in both idiopathic or secondary PCI and did not necessarily imply the presence of an associated underlying bowel disease [3]. In our case, the pneumoperitoneum was probably related to the gas-filled cyst rupture secondary to the abdominal trauma.

Ultrasonography can be used to detect PCI. This technique is more commonly applied to the pediatric patient in whom avoidance of ionizing radiation is preferred but in most cases it is hampered by the presence of tympanites [7].

The diagnosis of PCI can also be established by endoscopic ultrasound which demonstrates multiple hyperechoic air pockets with shadowing in the submucosal layer [8]. This examination can differentiate PCI from others lesions avoiding harmful endoscopic treatment.

Management of PCI is conditioned by the clinical and radiological presentation which is essentially related to the primary cause. Conservative approach is allowed in a stable patient with no signs of complications [2]. In the presence of predictive factors of pathologic PCI, namely transmural ischemia and bowel perforation, surgical procedure is required [9].

## 4 Conclusion

Management of PCI may be challenging particularly with the presence of pneumoperitoneum. Complications must be excluded before considering a conservative therapy. Therefore, PCI should be interpreted with relevance to the entire clinical context.

## Funding

The authors declare no source of funding for the study.

## Ethical approval

This study is exempt from ethical approval in the authors’ institution.

## Consent

Consent was obtained from the patient in question for publication of this case report and accompanying images. A copy of the written consent is available upon request.

## Author contribution

Belkhir Asma: Writing of the paper and scan interpretating.

Jrad Mariem: Assistant to the manuscript writing and scan interpretating.

Soudani Mariem: Assistant to the manuscript wrinting and scan interpretating.

Sebei Amine: Main surgeon involved in patient care.

Haddad Anis: Main surgeon involved in patient care.

Boukriba Seif: Contributor.

Frikha Wassim: Contributor.

Mizouni Habiba: Contributor.

## Registration of research studies

The authors don’t need to register this work.

## Guarantor

Dr. Belkhir Asma and Dr. Myriam Jrad, the corresponding authors of this manuscript accept full responsibility for the work and the conduct of the study, access to the data and controlled the decision to publish.

## Provenance and peer review

Not commissioned, externally peer-reviewed.

## Declaration of Competing Interest

The authors declare no conflict of interest.
